# Exploring the genetic etiology across the continuum of the general psychopathology factor: a Swedish population-based family and twin study

**DOI:** 10.1038/s41380-024-02552-2

**Published:** 2024-04-10

**Authors:** Yangjun Liu, Paul Lichtenstein, Roman Kotov, Henrik Larsson, Brian M. D’Onofrio, Erik Pettersson

**Affiliations:** 1 Department of Medical Epidemiology and Biostatistics, Karolinska Institutet, Stockholm, Sweden; 2 Department of Psychiatry and Behavioral Health, Stony Brook University, Stony Brook, NY USA; 3 School of Medical Sciences, Örebro University, Örebro, Sweden; 4 Department of Psychological and Brain Sciences, Indiana University, Bloomington, IN USA

**Keywords:** Psychiatric disorders, Depression

## Abstract

Psychiatric comorbidity can be accounted for by a latent general psychopathology factor (p factor), which quantifies the variance that is shared to varying degrees by every dimension of psychopathology. It is unclear whether the entire continuum of the p factor shares the same genetic origin. We investigated whether mild, moderate, and extreme elevations on the p factor shared the same genetic etiology by, first, examining the linearity of the association between p factors across siblings (*N* = 580,891 pairs). Second, we estimated the group heritability in a twin sample (*N* = 17,170 pairs), which involves testing whether the same genetic variants influence both extreme and normal variations in the p factor. In both samples, the p factor was based on 10 register-based psychiatric diagnoses. Results showed that the association between siblings’ p factors appeared linear, even into the extreme range. Likewise, the twin group heritabilities ranged from 0.42 to 0.45 (95% CI: 0.33–0.57) depending on the thresholds defining the probands (2–3.33 SD beyond the mean; >2 SD beyond the mean; >4.33 SD beyond the mean; and >5.33 SD beyond the mean), and these estimates were highly similar to the estimated individual differences heritability (0.41, 95% CI: 0.39–0.43), indicating that scores above and below these thresholds shared a common genetic origin. Together, these results suggest that the entire continuum of the p factor shares the same genetic origin, with common genetic variants likely playing an important role. This implies, first, genetic risk factors for the aspect that is shared between all forms of psychopathology (i.e., genetic risk factors for the p factor) might be generalizable between population-based cohorts with a higher prevalence of milder cases, and clinical samples with a preponderance of more severe cases. Second, prioritizing low-cost genome-wide association studies capable of identifying common genetic variants, rather than expensive whole genome sequencing that can identify rare variants, may increase the efficiency when studying the genetic architecture of the p factor.

## Introduction

Both cross-sectional and longitudinal studies have shown that psychiatric disorders often co-occur [1–4], and the shared variance among psychiatric conditions can be explained by a latent general psychopathology factor known as the “p factor” [5–9]. The p factor conceptually parallels the widely used general factor of intelligence (“g factor”) and reflects a spectrum of psychopathology severity where higher scores on the p factor indicate a greater liability toward multiple psychiatric diagnoses [10]. Family, twin, and genomic studies suggest that the p factor has a partly genetic basis [6, 11–17]. For instance, the heritability of the p factor based on twin studies is estimated at 43–60% [11, 16, 18–22], and the single nucleotide polymorphism (SNP)-based heritability from genomic studies is estimated at 16–38% [12, 23, 24].

Nevertheless, recent genomic studies have found low to moderate correlations between genetic risks for milder versus more severe psychiatric conditions. This indicates that mild versus extreme elevations on the p factor, in turn, might have distinct genetic etiologies. For instance, one study observed that whereas a total psychiatric problem score (a proxy for the p factor) was highly correlated with genetic risk for common psychiatric problems, the correlations were low with genetic risk for rare psychiatric conditions such as schizophrenia [25]. On a similar note, another study that jointly analyzed genetic risk for both common and rare psychiatric conditions identified two moderately correlated dimensions, the first of which captured genetic risk for common conditions (e.g., depression), and the second of which captured genetic risk for rarer forms of serious mental illness (e.g., schizophrenia) [26]. However, to date, no study has formally examined whether mild and severe elevations on the p factor share the same genetic etiology.

Clarifying whether genetic influences are the same across the continuum of the p factor could provide valuable insights for future psychiatric genetic research. When studying the genetic architecture of psychiatric disorders, cases can be recruited based on medical records or structured clinical interviews. These approaches have the advantage of capturing individuals with severe psychopathology, but the diagnostic process for cases can be time-consuming and costly, often resulting in a limited sample size. Recently, using data from population-based cohorts or health registers have become increasingly popular in psychiatric genetics research, which may accelerate the genetic discoveries due to large sample size and data availability. However, a critical concern is whether the preponderance of mild cases in such samples provides accurate information on genetic risk variants present in more severe cases.

In this study, we used Swedish national health register data and employed two approaches to investigate whether mild, moderate, and extreme elevations on the p factor shared the same genetic etiology. First, we examined the shape of the association between the p factors across siblings. If the same genetic variants were to contribute to all levels of the p factor (i.e., if it were a quantitative trait), then the association across siblings ought to be linear throughout. On the other hand, if different genetic variants were to contribute to mild versus extreme levels (i.e., if the extreme end were qualitatively different), then the association across siblings ought to be positive in the mild range but closer to null at the extremes (i.e., follow an inverted U-shaped pattern). As the latter pattern appears to explain the familial aggregation of the g factor (i.e., whereas mild intellectual disability exhibits high familial aggregation, extreme intellectual disability appears considerably less familial) [27, 28], we additionally conducted a negative control analysis by examining the association between different severity levels of intellectual disability and the p factor across siblings.

Second, as sibling associations can be attributed to genetics or shared environments or both, we additionally used twin data to decompose familial associations into that which could be attributed to genetics versus environmental factors. Furthermore, we estimated the group heritability using a DeFries–Fulker (DF) extremes analysis, which is based on the differential regression to the mean of the population in monozygotic and dizygotic twins [29, 30]. If individuals who are exposed to co-twins with extreme elevations on the p factor score above the population mean themselves, and this effect is more pronounced in monozygotic compared to dizygotic co-twins, it implies that extreme elevations on p factor is at least partially genetically influenced. A significant group heritability estimate implies that extreme and normal variations in the p factor are heritable and there is a genetic link between them [29–31]. In addition, if extreme and normal variations in the p factor share the same etiology, then the group heritability (*h*_g_^2^) and individual differences heritability (*h*^2^) are expected to be similar [29–31].

## Methods

### Participants

The source population for this study consisted of all individuals born in Sweden between January 1, 1980 and December 31, 1999 who had not died or emigrated before the end of the follow-up on December 31, 2013. We extracted data from the Swedish Medical Birth register, the Multi-Generation Register, the National Patient Register, and the National Crime Register. All registers were linked via the unique personal identification number assigned to each Swedish resident at birth.

We identified two samples. The first sample included the oldest full-sibling pair within each family (*N* = 580,891 pairs), with a mean age of 24.1 years (SD, 5.1; range, 14.1–34.0) at the end of the follow-up. The second sample consisted of 22,682 twin pairs, and after excluding 5512 pairs without zygosity information, the final sample comprised 17,170 pairs, including 5133 monozygotic (MZ) and 12,037 dizygotic (DZ) twin pairs. Zygosity was determined by being of opposite sex, DNA information, or a validated algorithm based on five questions concerning twin similarity (with a probability of correct classification ≥95%) [32]. The mean age of this twin sample at the end of the follow-up was 22.5 years (SD, 5.5; range, 14.1–34.0).

This study was approved by the Regional Ethical Review Board in Stockholm, Sweden. Informed consent was obtained from the twin sample but was not required for de-identified register data by law.

### Measures

We derived the p factor from the following 10 diagnoses assigned by psychiatrists after contact with the in- or outpatient psychiatric services: anxiety spectrum disorder (anxiety, obsessive-compulsive disorder, and/or post-traumatic stress disorder), depression, bipolar disorder, eating disorder, drug misuse, alcohol abuse, attention deficit hyperactivity disorder (ADHD), autism, tics, and schizophrenia (containing schizoaffective disorder). Supplementary Table 1 presents related International Classification of Diseases (ICD) codes.

#### Exposure

The exposure was older siblings’ observed total diagnostic sum score, which served as a proxy for the latent p factor. We turned the sum score into binary dummy codes, whereby each p sum score value was compared to a reference group with p sum score equal to 0 (i.e., 0 vs. 1; 0 vs. 2; etc.). The dummy-coding allowed for examining if the associations between the siblings increased in a linear fashion, even at very high scores (i.e., it allowed for investigating potential non-linearity).

#### Outcome

The outcome was the younger siblings’ observed total diagnostic sum score. To examine how associated the observed diagnostic sum score was with the corresponding latent p factor, we estimated its reliability, that is, how much variance in the sum score was accounted for by the latent p factor.

To derive the latent p factor, we applied exploratory structural equation modeling (ESEM) to the 10 psychiatric diagnoses [33]. We decided on the number of factors to extract based on scree plot [34], and then rotated the factors toward one general and several uncorrelated specific factors using the Direct Schmid–Leiman transformation [35]. This way, the general factor (p factor) captured the shared variance among all psychiatric diagnoses, whereas the specific factors captured the variance unique to subsets of psychiatric disorders over and above the p factor.

Because the factor indicators were binary diagnoses, we used Item Response Theory (IRT) to estimate how much variance in the total sum score was accounted for by the latent p factor (i.e., its reliability). IRT reliability estimates differ in two ways from those based on Classical Test Theory (which is suitable for continuously distributed factor indicators). First, IRT reliability is conditional on the latent score (e.g., reliability could be high for individuals who are above the latent mean, but low for individuals who are below the latent mean). Second, IRT reliability estimates are usually expressed in a scale-dependent fashion (unlike classical reliability estimates that are commonly expressed as a scale-free R^2^). To facilitate interpretability, we translated the scale-dependent IRT reliability estimate into a conditional Classical Test Theory estimate, such that the conditional reliability was expressed as a scale-free R^2^ [36]. An R^2^ above 0.70 (i.e., that the latent factor accounted for at least 70% of the variation in the corresponding sum score) is generally considered acceptable [37].

To ensure that the sum score of the younger and older siblings captured the same underlying construct, we tested whether the factor loadings were invariant in the younger and older siblings in two ways. First, we fit the aforementioned latent factor model within a two-group model framework (with one group for the younger siblings, and one for the older siblings), in which we allowed the latent factor loadings to vary between groups versus being constrained to equality. We then compared the difference in model fit (using the Comparative Fit Index, CFI, and Root Mean Square Error of Approximation, RMSEA) between the more constrained (i.e., where the loadings were constrained to equality) versus less constrained model (i.e., the model where the loadings were allowed to vary between groups). Based on simulations, Cheung and Rensvold recommended that a ΔCFI < 0.01 was inadequate to conclude that two nested models differed [38]. Second, using the less constrained model in which the loadings were allowed to vary, we examined the similarity in the factor loadings by computing the factor congruence coefficient, with values above 0.95 implying that two factors can be considered equal [39].

### Statistical analyses

#### Estimating the association between the exposure and outcome

We regressed the younger siblings’ p sum score onto the older siblings’ dummy-coded p sum score. As the exposure was binary (e.g., 0 vs. 1; 0 vs. 2; etc.), the ensuing betas correspond to mean differences in the younger siblings’ p sum score for each additional diagnoses in the older sibling. In addition to visually examining whether the associations appeared linear into the extreme, we also conducted a linear-by-linear trend test. This test is more suitable than adding a quadratic term in the regression when the exposure is a categorical variable. A significant *p*-trend value rejects the null hypothesis that the trend is non-linear [40]. All regressions included the younger siblings’ age as a covariate.

#### Negative control analysis

Past research has shown that whereas mild intellectual disability runs in families, severe intellectual disability seldom does (presumably because it is primarily caused by rare mutations or environmental factors such as traumatic brain injury unique to only one sibling) [27, 41]. Given that past research has shown that the p and g factors are inversely associated [42–45], if the p factor were mainly attributed to common genetic variants, then one might expect that it should be associated with mild but not with severe and profound intellectual disability. Therefore, as a negative control condition, we examined the familial coaggregation between the p factor and diagnoses of intellectual disability of varying degrees of severity. Specifically, we regressed younger siblings’ p factor onto the older siblings’ intellectual disability diagnosis, where mild (2–3.33 standard deviations [SD] below the g factor mean), moderate (3.33–4.33 SD below the g factor mean), and severe-profound (>4.33 SD below the g factor mean) intellectual disability were compared to a reference group without intellectual disability [27, 28, 46].

#### DF extremes analysis and twin heritability

We first computed the observed p sum scores for both twins. We then used a DF extremes analysis and a classical twin model to estimate the group heritability and individual differences heritability of these p sum scores, respectively. The DF analysis tests whether extreme and normal variations in the p factor are genetically linked [29–31]. We detail this approach in the Supplementary Method. Briefly, estimating group heritability involves identifying twins who score above a cut-off (i.e., probands), and then estimating the degree to which the means of their co-twins regress toward the population mean. If the mean of DZ co-twins regress further to the population mean than that of MZ co-twins, it would imply that p sum scores both above and below the specified cut-off are genetically linked. To facilitate comparison with the g factor, we used the same cut-offs as those for intellectual disability to define the proband groups, namely mild (2–3.33 SD above the p sum score mean), mild-profound (>2 SD above the p sum score mean), severe-profound (>4.3 SD above the p sum score mean), and profound (> 5.33 SD above the p sum score mean). In addition, if the group heritability estimates (*h*_g_^2^) are similar to those of the individual differences heritability (*h*^2^), this further suggests that p sum scores above and below the specified threshold likely have the same etiology [29, 30]. Therefore, we also applied the classical twin model to decompose the variance of the p sum score into additive genetic effects (A), shared environment effects (C), and nonshared environment effects (E) [47], and compared the individual differences heritability to the group heritability estimates.

### Sensitivity analyses

We conducted five sensitivity analyses to examine the robustness of the findings. First, we regressed the younger siblings’ latent p onto the older siblings’ dummy-coded observed p sum score (see Supplementary Fig. 1 for model diagram). The advantage of this approach was two-folded. Measurement error in the outcome can generate larger standard errors. As the latent factor model is estimated to have perfect reliability, this could lead to smaller standard errors. In addition, given the multidimensional nature of the psychiatric conditions, the sum score is likely not only associated with the latent p factor, but also with the specific factors to a smaller degree. In contrast, the latent p is fixed to be uncorrelated with the specific factors, such that the association between the observed p sum score and a latent p cannot be confounded by variance attributed to specific psychopathology factors.

Second, we performed a modified familial coaggregation analysis by expanding the number of conditions used to derive the p factor from 10 to 15. The motivation for this sensitivity analysis was to allow for a more fine-grained measurement model. However, the downside was that the number of indicators for each specific psychiatric factor was uneven. In particular, there were more indicators for the internalizing factor, such that some might end up with a high p sum score by having several anxiety-related diagnoses. Specifically, we decomposed the anxiety spectrum disorder into three separate diagnoses (anxiety, obsessive-compulsive disorder, and post-traumatic stress disorder), separated schizoaffective disorder from the schizophrenia diagnosis, and included oppositional defiant disorder and court convictions of violent and/or property crimes (e.g., homicide and theft) [48] to capture a broader range of externalizing behaviors. The ICD codes for the additional psychiatric diagnoses can be found in Supplementary Table 2.

Third, to examine whether the associations between the p sum scores across family members might be impacted by rare deleterious mutations or severe environmental factors such as traumatic brain injury, we excluded sibling and twin pairs in which at least one member of each pair had diagnoses of severe or profound intellectual disability, and then we re-ran the familial coaggregation analyses and DF extremes analysis.

Fourth, aside from the p sum score, we also used p factor scores as exposures and outcomes. Whereas sum scores create a scale by applying unit weights to each indicator (e.g., indicator 1, 2, 3, etc., are simply summed into a scale), factor scores allow the weights to vary (e.g., indicator 1 might contribute 0.5 units, indicator 2 might contribute 0.75 units, etc., to the scale score). Both approaches have their respective advantages [49–52].

Fifth, as some disorders might have a later age of onset, we re-ran the models in a subsample in which the participants were 28–34 years old.

Data were analyzed from February 2022 to December 2022 using software SAS 9.4 [53], Mplus 8.3 [54], and R 4.0.5 [55] with GPArotation [56] package.

## Results

### Latent p factor

The first five eigenvalues for the 10 psychiatric diagnoses were 4.82, 1.39, 0.97, 0.77, and 0.49. Based on the scree plot, we extracted three factors, which fit well (Table 1). We then rotated them to one general factor (p factor) and three specific factors. Table 1 displays that all psychiatric diagnoses loaded positively on the p factor, with an average loading of 0.55 (range: 0.35–0.68). The three specific factors captured internalizing (e.g., anxiety and depression), substance misuse (e.g., drug misuse and alcohol abuse), and neurodevelopmental (e.g., ADHD and autism) conditions. The model fit deteriorated only marginally (ΔCFI = 0; ΔRMSEA = 0.002) when constraining the loadings to equality between siblings (vs. allowing them to differ between siblings), and the factor congruence coefficients between the siblings equaled 0.99–1.00, indicating that the latent factor model replicated across the siblings.

**Table 1:** Exploratory structural equation modeling of 10 psychiatric disorders.

Psychiatric diagnosis	Rotation: Direct Schmid–Leiman			
	General p factor	Specific internalizing factor	Specific substance misuse factor	Specific neurodevelopmental factor
Anxiety spectrum disorder	**0.67**	**0.51**	0.10	0.05
Depression	**0.68**	**0.62**	0.02	0.03
Bipolar disorder	**0.61**	**0.42**	0.12	0.07
Eating disorder	**0.35**	**0.56**	-0.09	-0.12
Drug misuse	**0.68**	0.13	**0.60**	-0.05
Alcohol abuse	**0.47**	0.14	**0.42**	-0.09
ADHD	**0.67**	0	0.11	**0.56**
Autism	**0.52**	0.12	-0.15	**0.55**
Tics	**0.42**	-0.01	-0.10	**0.53**
Schizophrenia	**0.49**	0.30	0.11	0.08

Note. Loadings greater than 0.30 are bolded. Anxiety spectrum disorder includes anxiety, obsessive-compulsive disorder, and post-traumatic stress disorder. ADHD: attention deficit hyperactivity disorder. Schizophrenia contains schizoaffective disorder. Model fit: Root Mean Square Error of Approximation (RMSEA) = 0.006, 90% confidence interval (CI) 0.006-0.007; Comparative Fit Index (CFI) = 0.999; Tucker-Lewis Index (TLI) = 0.996; Standardized Root Mean Square Residual (SRMR) = 0.034; and χ^2^(18) = 418.331, *p* < 0.001.

### Observed p sum score

**Fig. 1:**
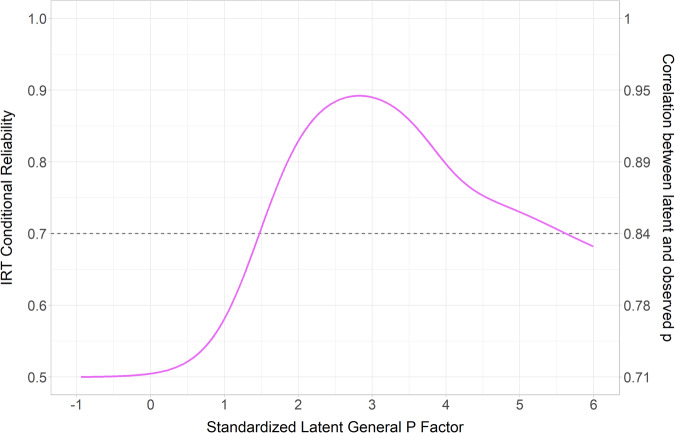
Item response theory (IRT) conditional reliability of the p sum score. Note. Reliability is the variance explained in the observed p sum score by the latent p factor. The reliability peaked close to 0.9 when the standardized latent p factor was 2.5–3 SD above the mean. For values more than 4 SD above the mean, which approximately fall in the same range as severe-profound ID, the reliability of the observed p sum score was estimated at 0.8 to 0.7, which we deemed acceptable. Overall, the reliability of the observed p sum score was good to great across the range that pertained to our research question.

The observed p sum score ranged from 0 to 9 (Supplementary Table 3 displays its tabulation, and Supplementary Fig. 2 shows the distribution), with a mean of 0.23 (SD = 0.68). The estimated conditional reliability of the p sum score is displayed in Fig. 1. Reliability exceeded 0.70 among individuals scoring between 1.5 and 5.5 standard deviations above the mean on the latent p factor, indicating that the sum score was adequately reliable within the range that pertained to our research question.

### Sibling aggregation of the p factor and negative control results

**Fig. 2:**
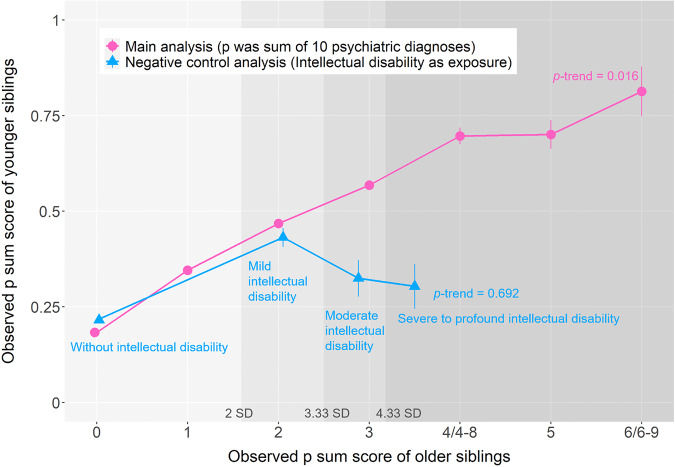
The mean of p sum score of younger siblings by p sum score and intellectual disability of older siblings. Note. The numbers (2 SD, 3.33 SD, and 4.33 SD) above the x axis represent number of standard deviation (SD) beyond the mean of the observed p sum score. Based on the deviations, the four regions with gradient shadings represent the severity of p factor in reference to the general population and correspond to “without”, “mild” (2–3.33 SD beyond the mean), “moderate” (3.33–4.33 SD beyond the mean), and “severe-profound” (>4.33 SD beyond the mean) intellectual disabilities, respectively. Observed p sum score was calculated as individuals’ total number of psychiatric diagnoses. The *p-*trend value was from linear-by-linear trend test (a significant *p*-trend value rejects the null hypothesis that the trend is non-linear). Bars represent 95% confidence intervals.

Older siblings’ p sum scores predicted younger siblings’ p sum scores, and this association appeared roughly linear even into the extreme (Fig. 2, Supplementary Table 4). Furthermore, the linear-by-linear trend test rejected the null hypothesis that the association was non-linear (*p-trend* = 0.016).

By contrast, in the negative control analysis in which the younger siblings’ p sum score was regressed on the older siblings’ intellectual disability of different severity levels, the association appeared distinctly non-linear (Fig. 2; Supplementary Table 5). That is, individuals whose siblings had a diagnosis of mild intellectual disability also had elevated scores on the p sum score (β = 0.22; 95% CI: 0.19–0.24), whereas the p sum scores were lower for those who were exposed to a sibling with moderate (β = 0.11; 95% CI: 0.06–0.16) or severe-profound intellectual disability (β = 0.09; 95% CI: 0.03–0.15). The linear-by-linear trend test did not reject the null hypothesis that the association was non-linear (*p-trend* = 0.69).

### DF extremes analysis and twin heritability

For the twin sample, the observed p sum score ranged from 0 to 7, with mean 0.19 and SD 0.60. The DF extremes analysis estimated the group heritability between 0.42 and 0.45 (95% CI range, 0.33–0.56) for different thresholds defining probands (Table 2a), which indicates genetic links between extreme and non-extreme p sum scores.

**Table 2:** Results of Defries–Fulker extremes analysis and classical twin model.

a. Defries–Fulker extremes analysis				
Threshold for observed p sum score used to define the proband group	Extremes analyses of the p factor			
	Extreme group correlations (Number of probands)		Extremes analysis estimates (95% CI)	
	MZ	DZ	Group heritability (*h*_*g*_^2^)	Nonshared environment
=2 (corresponding to mild ID)	0.44 (270)	0.17 (711)	0.44 (0.36, 0.51)	0.56 (0.48, 0.64)
≥2 (corresponding to mild-profound ID)	0.43 (434)	0.14 (1126)	0.43 (0.37, 0.48)	0.57 (0.51, 0.63)
≥3 (corresponding to severe-profound ID)	0.42 (164)	0.11 (415)	0.42 (0.35, 0.49)	0.58 (0.51, 0.66)
≥4 (corresponding to profound ID)	0.45 (53)	0.11 (139)	0.45 (0.33, 0.56)	0.55 (0.43, 0.67)
**b. Classical twin model**				
	**Analysis of the full range of the p factor**			
	**Intraclass correlations (95% CI)**		**Model-fitting estimates**^**a**^** (95% CI)**	
	**MZ**	**DZ**	**Heritability (*h***^**2**^)	**Nonshared environment**
Observed p sum score	0.45 (0.43, 0.47)	0.14 (0.12, 0.16)	0.41 (0.39, 0.43)	0.59 (0.57, 0.61)

Note. *MZ* monozygotic twins, *DZ* dizygotic twins, *ID* intellectual disability.

Based on the number of standard deviations (=0.60) beyond the mean value (=0.19), observed p sum score =2, ≥2, ≥3, and ≥4 correspond to mild ID (2–3.33 SD deviations above the mean), mild-profound ID (>2 SD deviations above the mean), severe-profound ID (>4.33 SD deviations above the mean), and profound ID (>5.33 SD deviations above the mean), respectively. Observed p sum score was calculated as individuals’ total number of psychiatric diagnoses.

^a^Because the DZ correlation was less than half the MZ correlation, there was no indication of shared environmental effects (C) and so we omitted it from the model.

The intraclass correlations for the p sum score were 0.45 for MZ twins and 0.14 for DZ twins (Table 2b). Because the DZ correlation was less than half the MZ correlation, there was no evidence of shared environment effects, which indicates that the sibling aggregation was primarily attributable to genetics. The estimated individual differences heritability was 0.41 (95% CI, 0.39–0.43), which was highly similar to the group heritability. This further suggests that the same genetic factors appear to influence both extreme and normal variations in the p sum score.

### Sensitivity analyses

First, when regressing the younger siblings’ latent p onto the older siblings’ dummy-coded observed p sum score, the results remained very similar to when using an observed p sum score as the outcome (Supplementary Tables 6 and 7; Supplementary Fig. 3). This indicates that the main results likely were not overly influenced by outcome measurement error or contaminated by specific psychopathology variance. Second, in the analysis that included 15 conditions (i.e., 14 psychiatric diagnoses plus criminality) to derive the p factor, the first five Eigenvalues were 6.49, 1.61, 1.43, 1.11, and 0.69. We thus extracted four factors that fit the data well (Supplementary Table 8). Familial coaggregation analysis generated similar results as the analysis deriving the p factor from 10 psychiatric diagnoses (Supplementary Fig. 3), suggesting that the results were robust when using a more fine-grained measurement model. Third, the familial coaggregation analysis of siblings and DF extremes analysis of twins, after excluding pairs where at least one member had severe or profound intellectual disability, yielded similar results (Supplementary Tables 9–11), indicating that the results did not appear attributable to the etiology of severe intellectual disability. Fourth, when regressing the younger siblings’ standardized p factor score onto the older siblings’ standardized p factor score, the results remained highly similar (Supplementary Table 12; Supplementary Fig. 4), suggesting that the results were not overly influenced by whether we used unit- or non-unit weights when computing the observed score. Fifth, as outlined in Supplementary Fig. 3, highly similar results emerged when we only analyzed siblings who were 28–34 years old, suggesting that the age range in the original sample seemed unlikely as a source of bias.

## Discussion

We used 10 psychiatric conditions to estimate a latent p factor, which quantifies the variance that is shared to varying degrees by every dimension of psychopathology. We observed that mild, moderate, and extreme elevations on this p factor were familial and the reason for this appeared genetic rather than environmental. Moreover, the whole range of the p factor appears to be part of the same underlying continuum affected by the same genetic factors.

The continuity in the genetic origin of the p factor indicates that genetic variants associated with mild elevation on the p factor are also expected to contribute to moderate and extreme elevations on the p factor, and vice versa. Thus, findings from population-based cohorts, which predominantly consist of milder cases, might be generalizable to clinical cases that typically exhibit more severe symptoms. Thus, molecular genetic studies might benefit from using large population-based samples (e.g., the UK Biobank and Nordic national health register data), which could enhance statistical power.

The entire continuum of the p factor appeared to share the same genetic etiology, and it has strong associations with mild but not with severe-profound intellectual disability. One speculation is that the shared variance among psychiatric disorders (i.e., the p factor) might be predominantly influenced by common genetic variants with small effects, which is consistent with previous studies. For instance, psychiatric polygenic risk scores have been found to predict the p factor [15, 57–59], and two specific loci appear associated with the total psychiatric problem score, a proxy for the p factor [25]. Also, the SNP-based p factor heritability is estimated at 16–38% [12, 23, 24]. Together, these results imply that when studying the genetic architecture of the p factor, focusing primarily on low-cost genome-wide association studies capable of detecting common variants, rather than expensive whole-genome sequencing that identify rare variants, may lead to increased efficiency and substantial advancements. Nevertheless, this does not exclude the influence of rare variants, as they might explain the missing heritability [60]. Moreover, rare variants could have different ranges of penetrance and expressivity, which could also result in continuous phenotypes in populations [61]. Additionally, prior research has found that rare copy number variants were weakly but significantly associated with the p factor [62].

These results might also bear on the inverse association between the g and p factors [42–45]. To the extent that mild intellectual disability captures the low end of cognitive ability, this implies that the overlap between g and p might be attributed to common genetic variants, rather than to deleterious rare genetic variants that are often linked to severe intellectual disability. In contrast, we observed almost no attenuation in the association between intellectual disability and the specific neurodevelopmental factor regardless of the severity of the intellectual disability (Supplementary Table 7), suggesting that both common and rare genetic variants might contribute to conditions such as autism, in line with past genomic studies [63, 64].

To the best of our knowledge, this study is the first one using population-based family data to examine the continuum of the genetic etiology of the p factor. The large sample size allowed us to examine the etiology at the extreme end of the p factor spectrum with relatively high precision. Nevertheless, the results should be interpreted in light of some limitations. First, we used observed p sum score, which might lead to underestimated associations and increased standard errors due to measurement error. However, the reliability of the observed p sum score was estimated as adequate throughout the range of interest, and using a latent p factor (i.e., which is assumed to be free from measurement error) as the outcome generated similar results, such that unreliability seems unlikely to explain the linear familial association. Second, the observed p sum score exhibited a positively skewed distribution, which might have led to bias in the main analyses where we used p sum score as both exposure and outcome. However, similar results emerged when we regressed latent p factor onto p sum score, in which skewness is less likely to bias the results. Additionally, skewness might have slightly inflated the estimates of DF group heritability [65]. Nevertheless, DF extremes analysis appears robust to severely skewed data [65], such that the potentially slight overestimation of group heritability seems unlikely to bias the overall conclusion. Third, we derived the p factor from register-based clinical diagnoses, which tend to capture more severe cases and may be less reliable than structured clinical interviews. However, the genetic correlation between psychiatric diagnoses obtained through structured clinical interviews, and those from primary care or specialist care registries, is nearly perfect [66]. Fourth, the average age of the study samples was around 24 years old, such that some might not have lived long enough to attain the more severe diagnoses. However, similar results emerged when we analyzed a subsample who were 28–34 years old, suggesting that this limitation likely does not impact our overall conclusion. Fifth, we only relied on pairs of siblings and twins to infer the genetic architecture of the p factor. Future research would benefit from applying genomic approaches, which can directly measure both common and rare genetic variants.

In conclusion, in this study, the entire continuum of the p factor appeared to share the same genetic etiology, with common genetic variants likely playing an important role. These findings indicate that genetic risk factors for the aspect that is shared between all forms of psychopathology (i.e., genetic risk factors for the p factor) might be generalizable between population-based cohorts with a higher prevalence of milder cases, and clinical samples with a preponderance of more severe cases. Additionally, prioritizing low-cost genome-wide association studies capable of identifying common genetic variants, rather than expensive whole genome sequencing that can identify rare variants, may increase the efficiency when studying the genetic architecture of the p factor.

## Supplementary information

Supplementary materials

## Data Availability

Data used for these analyses are available from the corresponding author upon reasonable request.

## Code availability

Code used for these analyses are available from the corresponding author upon reasonable request.
