# Supplementary material for: Exploring the genetic etiology across the continuum of the general psychopathology factor: a Swedish population-based family and twin study
Source: Mol Psychiatry. 2024 Apr 10;29(10):2921–8. doi: 10.1038/s41380-024-02552-2 (PMC11449779; doi:10.1038/s41380-024-02552-2)
Supplement: Supplementary file 1 — Supplementary materials [file 41380_2024_2552_MOESM1_ESM.docx]

**Supplementary Online Content**

**Article title**

Exploring the genetic etiology across the continuum of the general psychopathology factor: A Swedish population-based family and twin study

**Authors**

Yangjun Liu, MD, PhD^1^; Paul Lichtenstein, PhD^1^; Roman Kotov, PhD^2^; Henrik Larsson, PhD^1,3^; Brian M. D’Onofrio, PhD^1,4^; Erik Pettersson, PhD^1^

^1^Department of Medical Epidemiology and Biostatistics, Karolinska Institutet, Stockholm, Sweden

^2^Department of Psychiatry and Behavioral Health, Stony Brook University, Stony Brook, New York, United States

^3^School of Medical Sciences, Örebro University, Örebro, Sweden

^4^Department of Psychological and Brain Sciences, Indiana University, Bloomington, Indiana, United States

**Supplementary Content**

**Supplementary Method.** DeFries-Fulker Extremes Analysis

**Supplementary Table 1.** International Classification of Diseases (ICD) codes for the psychiatric diagnoses included in the main analysis

**Supplementary Table 2.** International Classification of Diseases (ICD) codes for additional psychiatric diagnoses included in the second sensitivity analysis

**Supplementary Table 3.** Distribution of the observed p sum score of older siblings

**Supplementary Table 4.** Coefficients for familial coaggregation analysis regressing younger siblings’ observed p sum score onto older siblings’ binned observed p sum score (main analysis)

**Supplementary Table 5.** Coefficients for familial coaggregation analysis regressing younger siblings’ observed p sum score onto older siblings’ intellectual disability (negative control analysis for the main analysis)

**Supplementary Table 6.** Coefficients for familial coaggregation analysis regressing younger siblings’ latent p factor and specific psychiatric factors onto older siblings’ binned observed p sum score (sensitivity analysis 1)

**Supplementary Table 7.** Coefficients for familial coaggregation analysis regressing younger siblings’ latent p factor and specific psychiatric factors onto older siblings’ intellectual disability (negative control analysis for sensitivity analysis 1)

**Supplementary Table 8.** Exploratory structural equation modeling of 15 conditions (sensitivity analysis 2)

**Supplementary Table 9.** Exploratory structural equation modeling of 10 psychiatric disorders after excluding sibling pairs where at least one member had severe and profound intellectual disability (sensitivity analysis 3)

**Supplementary Table 10.** Coefficients for familial coaggregation analysis regressing younger siblings’ latent p factor and specific psychiatric factors onto older siblings’ binned observed p sum score after excluding sibling pairs where at least one member had severe and profound intellectual disability (sensitivity analysis 3)

**Supplementary Table 11.** Results of classical twin model and Defries-Fulker extremes analysis after excluding twin pairs where at least one member had severe and profound intellectual disability (sensitivity analysis 3)

**Supplementary Table 12.** Coefficients for familial coaggregation analysis regressing younger siblings’ standardized p factor score onto older siblings’ binned standardized p factor score (sensitivity analysis 4)

**Supplementary Figure 1.** Schematic of structural equation modeling diagram using latent p factor as outcome and p sum score as exposure

**Supplementary Figure 2.** Distribution of the observed p sum score of older siblings

**Supplementary Figure 3.** Comparison of familial coaggregation results regressing younger siblings’ latent p factor onto older siblings’ binned observed p sum score (sensitivity analyses 1, 2, and 5) and intellectual disability (negative control analysis)

**Supplementary Figure 4.** Comparison of familial coaggregation results regressing younger siblings’ standardized p factor score onto older siblings’ standardized p factor score (sensitivity analyses 4) and intellectual disability (negative control analysis)

**Supplementary References**

**Supplementary Method.** DeFries-Fulker Extremes Analysis

DeFries-Fulker Extremes Analysis is based on the differential regression to the mean of the population using identical (monozygotic, MZ) and fraternal (dizygotic, DZ) twins [1, 2, 3]. We ascertained probands whose observed p sum scores were above the threshold and their cotwins. Because there is no acknowledged cut-off for extreme p sum score, in this study, we used several exploratory thresholds to define the probands. The mean of the observed p sum scores for the probands, MZ cotwins, DZ cotwins, and the unselected population were expected to be different, and the amount of the differential regression of MZ and DZ cotwins’ means toward the unselected population mean suggests the extent to which the extreme p sum score is due to genetic factors [1, 2, 3]. Moreover, after standardizing the probands’ mean to 1 and the population mean to 0, the transformed MZ and DZ cotwins’ means represent MZ and DZ correlations, respectively [1, 2, 3]. If the mean of DZ cotwins regress further than that of MZ cotwins to the population mean (i.e., if the MZ correlation is higher than the DZ correlation), it implies that the extreme p sum score is partially genetically influenced [1, 2, 3]. The extent to which genetic factors account for the mean difference between a selected proband group and the unselected population is labeled as group heritability (*h*_g_^2^) [1, 2, 3], and a significant group heritability indicates that p sum scores above and below the threshold are heritable and there is a genetic link between them [1, 2, 3]. The group heritability (*h*_g_^2^) is calculated as *h*_g_^2^ = 2 * (transformed MZ cotwins’ mean - transformed DZ cotwins’ mean) [1, 2, 3], and the nonshared environmental effect is estimated as 1 - transformed MZ cotwins’ mean [1, 2, 3]. Of note, the estimated group heritability should not be larger than the MZ correlation (i.e., the value of the transformed MZ cotwins’ mean); otherwise, it takes the value of the MZ correlation. The DeFries-Fulker Extremes Analysis could be conducted using multiple regression model, C = B_1_P + B_2_R + A, where C, P, R, and A represent the cotwins’ p sum score, probands’ p sum score, coefficient of the relationship (1 for MZ and 0.5 for DZ twin pairs), and regression constant, respectively [1, 2, 3]. The ratio B_2_/(probands’ mean score – population mean score) estimates the group heritability [1, 2, 3], and if the probands’ mean and population mean are transformed to 1 and 0, respectively, then the regression coefficient B_2_ estimates the group heritability directly [1, 2, 3]. In addition, if the probands with extreme p sum scores represent the high end of the p factor distribution, the group heritability (*h_g_*^2^) would be expected to be similar to the individual differences heritability (*h*^2^) that represents the extent to which genetic factors account for individual differences in the population [1, 2, 3].

**Supplementary Table 1.** International Classification of Diseases (ICD) codes for the psychiatric diagnoses included in the main analysis

| Disorder | ICD-8 (1969-1986) | ICD-9 (1987-1996) | ICD-10 (1997-2013) |
| --- | --- | --- | --- |
| Anxiety spectrum disorder | 300 (excl. 300.4)  307 | 300 (excl. 300E)  308, 309 | F40-45, F48 |
| Depression | 300.4 | 311; 300E | F32-F39 |
| Bipolar disorder | 296.0-296.3, 296.8, 296.9 | 296A-296E, 296W,  296X | F30, F31 |
| Eating disorder | 3065, 7840 | 307B, 307F | F50 |
| Drug misuse | 304 | 292, 304, 305X | F11-19 (excl. F11.5, F12.5, F13.5, F14.5, F15.5, F16.5, F17, F18.5, F19.5) |
| Alcohol abuse | 291, 303 | 291, 303, 305A | F10 (excl. F10.5) |
| Attention deficit hyperactivity disorder | / | 314 | F90 |
| Autism | / | 299 | F84 (excl. F84.2) |
| Tics | / | 307C | F95 |
| Schizophrenia | 295.0-295.4, 295.6,  295.7, 295.8, 295.9 | 295A-295E, 295G, 295H, 295W, 295X | F20, F25 |
| Mild intellectual disability | 311 | 317 | F70 |
| Moderate intellectual disability | 312 | 318A | F71 |
| Severe and profound intellectual disability | 313, 314 | 318B, 318C | F72-73 |

Note: ICD-8 and ICD-9 diagnoses were coded using the Swedish versions which are not fully consistent with the international versions. Missing in the ICD-8 is due to the non-existence of corresponding diagnoses.

**Supplementary Table 2.** International Classification of Diseases (ICD) codes for additional psychiatric diagnoses included in the second sensitivity analysis

| Disorder | ICD-8 (1969-1986) | ICD-9 (1987-1996) | ICD-10 (1997-2013) |
| --- | --- | --- | --- |
| Anxiety | 300 (excl. 300.3, 300.4) | 300 (excl. 300E, 300D) | F40-41, F44-45, F48 |
| Obsessive-compulsive disorder | 300.3 | 300D | F42 |
| Post-traumatic stress disorder | 307 | 308, 309 | F43 |
| Schizophrenia | 295.0-295.4, 295.6,  295.8, 295.9 | 295A-295E, 295G,  295W, 295X | F20 |
| Schizoaffective disorder | 295.7 | 295H | F25 |
| Oppositional defiant disorder | / | 312 | F91 |

Note: ICD-8 and ICD-9 diagnoses were coded using the Swedish versions which are not fully consistent with the international versions. Missing in the ICD-8 is due to the non-existence of corresponding diagnoses.

**Supplementary Table 3.** Distribution of the observed p sum score of older siblings

| **Observed p sum score** | **Number of individuals (Percentage %)** | |
| --- | --- | --- |
| 0 | | 498,939 (85.89) |
| 1 | | 48,692 (8.38) |
| 2 | | 20,026 (3.45) |
| 3 | | 84,62 (1.46) |
| 4 | | 32,88 (0.57) |
| 5 | | 1,115 (0.19) |
| 6 | | 300 (0.05) |
| 7 | | 63 (0.01) |
| 8 | | 5 (< 0.01) |
| 9 | | 1 (<0.01) |

**Supplementary Table 4.** Coefficients for familial coaggregation analysis regressing younger siblings’ observed p sum score onto older siblings’ binned observed p sum score (main analysis)

| Observed p sum score | Beta (95% Confidence interval) |
| --- | --- |
| 0 | 0 |
| 1 | 0.16 (0.16, 0.17) |
| 2 | 0.28 (0.28, 0.29) |
| 3 | 0.38 (0.37, 0.40) |
| 4 | 0.51 (0.49, 0.54) |
| 5 | 0.52 (0.48, 0.55) |
| 6 | 0.63 (0.56, 0.69) |

Note: Group without any psychiatric diagnosis (i.e., observed p sum score = 0) was the reference group.

**Supplementary Table 5.** Coefficients for familial coaggregation analysis regressing younger siblings’ observed p sum score onto older siblings’ intellectual disability (negative control analysis for the main analysis)

| Intellectual disability | Beta (95% Confidence interval) |
| --- | --- |
| Without intellectual disability | 0 |
| Mild intellectual disability | 0.22 (0.19, 0.24) |
| Moderate intellectual disability | 0.11 (0.06, 0.16) |
| Severe to profound intellectual disability | 0.09 (0.03, 0.15) |

Note: Group without intellectual disability was the reference group.

**Supplementary Table 6.** Coefficients for familial coaggregation analysis regressing younger siblings’ latent p factor and specific psychiatric factors onto older siblings’ binned observed p sum score (sensitivity analysis 1)

| Observed p sum score | Rotation: Direct Schmid-Leiman | | | |
| --- | --- | --- | --- | --- |
|  | p factor | Specific internalizing factor | Specific substance misuse factor | Specific neurodevelopmental factor |
| 0 | 0 | 0 | 0 | 0 |
| 1 | 0.38  (0.36, 0.39) | 0.06  (0.0.04, 0.08) | 0.10  (0.08, 0.13) | 0.22  (0.19, 0.24) |
| 2 | 0.55  (0.53, 0.57) | 0.15  (0.12, 0.18) | 0.08  (0.04, 0.11) | 0.33  (0.29, 0.36) |
| 3 | 0.69  (0.66, 0.72) | 0.15  (0.11, 0.19) | 0.18  (0.13, 0.23) | 0.37  (0.32, 0.41) |
| 4 | 0.82  (0.78, 0.87) | 0.20  (0.14, 0.26) | 0.23  (0.16, 0.31) | 0.39  (0.33, 0.46) |
| 5 | 0.83  (0.76, 0.91) | 0.15  (0.04, 0.26) | 0.28  (0.15, 0.41) | 0.40  (0.29, 0.52) |
| 6-9 | 0.94  (0.80, 1.07) | 0.20  (0.02, 0.37) | 0.32  (0.12, 0.53) | 0.41  (0.23, 0.60) |

Note: Group without any psychiatric diagnosis (i.e., observed p sum score = 0) was the reference group. Numbers in the parentheses are 95% confidence intervals.

**Supplementary Table 7.** Coefficients for familial coaggregation analysis regressing younger siblings’ latent p factor and specific psychiatric factors onto older siblings’ intellectual disability (negative control analysis for sensitivity analysis 1)

| Intellectual disability | Rotation: Direct Schmid-Leiman | | | |
| --- | --- | --- | --- | --- |
|  | p factor | Specific internalizing factor | Specific substance misuse factor | Specific neurodevelopmental factor |
| Without | 0 | 0 | 0 | 0 |
| Mild | 0.46  (0.40, 0.51) | -0.06  (-0.15, 0.02) | 0.06  (-0.04, 0.17) | 0.46  (0.38, 0.54) |
| Moderate | 0.31  (0.21, 0.41) | -0.25  (-0.45, -0.05) | 0.16  (-0.05, 0.37) | 0.40  (0.24, 0.57) |
| Severe to profound | 0.17  (0.02, 0.32) | 0.11  (-0.12, 0.33) | -0.34  (-0.65, -0.03) | 0.40  (0.21, 0.60) |

Note: Group without intellectual disability was the reference group. Numbers in the parentheses are 95% confidence interval.

**Supplementary Table 8.** Exploratory structural equation modeling of 15 conditions (sensitivity analysis 2)

|  | Rotation: Direct Schmid-Leiman | | | | |
| --- | --- | --- | --- | --- | --- |
|  | p factor | Specific internalizing factor | Specific externalizing factor | Specific neurodevelopmental factor | Specific psychotic factor |
| Anxiety | **0.66** | **0.51** | 0.09 | 0.03 | 0.04 |
| OCD | **0.51** | **0.44** | -0.21 | 0.22 | 0.06 |
| PTSD | **0.56** | **0.43** | 0.22 | 0.00 | -0.08 |
| Depression | **0.68** | **0.58** | 0.06 | 0.02 | 0.01 |
| Bipolar disorder | **0.64** | **0.36** | 0.10 | 0.02 | 0.18 |
| Eating disorder | **0.35** | **0.57** | -0.08 | -0.11 | -0.04 |
| Drug misuse | **0.68** | 0.11 | **0.53** | -0.04 | 0.07 |
| Alcohol abuse | **0.48** | 0.12 | **0.46** | -0.07 | -0.02 |
| Criminality | **0.33** | -0.05 | **0.39** | 0.05 | -0.06 |
| ODD | **0.53** | 0 | 0.25 | **0.47** | -0.19 |
| ADHD | **0.66** | 0.02 | 0.11 | **0.58** | -0.05 |
| Autism | **0.55** | 0.08 | -0.21 | **0.52** | 0.17 |
| Tics | **0.37** | 0.08 | -0.20 | **0.59** | -0.10 |
| Schizophrenia | **0.58** | 0.07 | -0.02 | -0.02 | **0.56** |
| Schizoaffective disorder | **0.65** | 0.09 | 0.01 | -0.15 | **0.69** |

Note. Loadings greater than 0.30 are bolded. OCD: obsessive-compulsive disorder; PTSD: post-traumatic stress disorder; ODD: oppositional-defiance disorder; ADHD: attention deficit hyperactivity disorder. Model fit: Root Mean Square Error of Approximation (RMSEA) = 0.006, 90% confidence interval (CI) 0.006-0.007; Comparative Fit Index (CFI) = 0.997; Tucker-Lewis Index (TLI) = 0.993; Standardized Root Mean Square Residual (SRMR) = 0.033; and ꭓ^2^(51) = 1241.639, p < 0.001.

**Supplementary Table 9.** Exploratory structural equation modeling of 10 psychiatric disorders after excluding sibling pairs where at least one member had severe and profound intellectual disability (sensitivity analysis 3)

| Psychiatric diagnosis | Rotation: Direct Schmid-Leiman | | | |
| --- | --- | --- | --- | --- |
|  | p factor | Specific internalizing factor | Specific substance misuse factor | Specific neurodevelopmental factor |
| Anxiety spectrum disorder | **0.67** | **0.51** | 0.10 | 0.06 |
| Depression | **0.68** | **0.62** | 0.02 | 0.03 |
| Bipolar disorder | **0.61** | **0.42** | 0.12 | 0.07 |
| Eating disorder | **0.35** | **0.56** | -0.09 | -0.12 |
| Drug misuse | **0.68** | 0.13 | **0.60** | -0.05 |
| Alcohol abuse | **0.47** | 0.14 | **0.42** | -0.09 |
| ADHD | **0.67** | 0 | 0.11 | **0.56** |
| Autism | **0.52** | 0.12 | -0.15 | **0.55** |
| Tics | **0.42** | -0.01 | -0.10 | **0.53** |
| Schizophrenia | **0.49** | 0.30 | 0.12 | 0.08 |

Note. This sample contained 579,902 sibling pairs. Loadings greater than 0.30 are bolded. Anxiety spectrum disorder includes anxiety, obsessive-compulsive disorder, and post-traumatic stress disorder. ADHD: attention deficit hyperactivity disorder. Schizophrenia contains schizoaffective disorder. Model fit: Root Mean Square Error of Approximation (RMSEA) = 0.006, 90% confidence interval (CI) 0.006-0.007; Comparative Fit Index (CFI) = 0.999; Tucker-Lewis Index (TLI) = 0.997; Standardized Root Mean Square Residual (SRMR) = 0.034; and ꭓ^2^(18) = 413.999, *p* < 0.001.

**Supplementary Table 10.** Coefficients for familial coaggregation analysis regressing younger siblings’ latent p factor and specific psychiatric factors onto older siblings’ binned observed p sum score after excluding sibling pairs where at least one member had severe and profound intellectual disability (sensitivity analysis 3)

| Observed p sum score | Rotation: Direct Schmid-Leiman | | | |
| --- | --- | --- | --- | --- |
|  | p factor | Specific internalizing factor | Specific substance misuse factor | Specific neurodevelopmental factor |
| 0 | 0 | 0 | 0 | 0 |
| 1 | 0.38  (0.36, 0.39) | 0.06  (0.0.04, 0.08) | 0.11  (0.08, 0.13) | 0.21  (0.19, 0.24) |
| 2 | 0.55  (0.53, 0.57) | 0.15  (0.12, 0.18) | 0.08  (0.04, 0.11) | 0.33  (0.29, 0.36) |
| 3 | 0.69  (0.66, 0.72) | 0.15  (0.12, 0.18) | 0.18  (0.13, 0.23) | 0.36  (0.32, 0.41) |
| 4 | 0.82  (0.78, 0.87) | 0.20  (0.13, 0.26) | 0.23  (0.16, 0.31) | 0.40  (0.33, 0.46) |
| 5 | 0.83  (0.76, 0.91) | 0.15  (0.04, 0.26) | 0.29  (0.16, 0.42) | 0.40  (0.28, 0.51) |
| 6-9 | 0.91  (0.78, 1.05) | 0.22  (0.04, 0.40) | 0.33  (0.12, 0.54) | 0.37  (0.18, 0.55) |

Note: This sample contained 579,902 sibling pairs. Group without any psychiatric diagnosis (i.e., observed p sum score = 0) was the reference group. Numbers in the parentheses are 95% confidence intervals.

**Supplementary Table 11.** Results of classical twin model and Defries-Fulker extremes analysis after excluding twin pairs where at least one member had severe and profound intellectual disability (sensitivity analysis 3)

| Table 9a. Defries-Fulker extremes analysis | | | | |  |
| --- | --- | --- | --- | --- | --- |
| Threshold for observed p sum score used to define the proband group | Extremes analyses of the p factor | | | | |
|  | Extreme group correlations  (Number of probands) | |  | Extremes analysis estimates  (95% CI) | |
|  | MZ | DZ |  | Group heritability (*h_g_*^2^) | Nonshared environment |
| = 2 (corresponding to mild ID) | 0.44 (268) | 0.16 (709) |  | 0.44 (0.36, 0.52) | 0.56 (0.48, 0.64) |
| ≥ 2 (corresponding to mild-profound ID) | 0.43 (432) | 0.14 (1122) |  | 0.43 (0.38, 0.49) | 0.57 (0.51, 0.63) |
| ≥ 3 (corresponding to severe-profound ID) | 0.42 (164) | 0.11 (413) |  | 0.42 (0.35, 0.50) | 0.58 (0.50, 0.65) |
| ≥ 4 (corresponding to profound ID) | 0.45 (53) | 0.11 (139) |  | 0.45 (0.33, 0.57) | 0.55 (0.43, 0.67) |
|  |  |  |  |  |  |
| Table 9b. Classical twin model | | | | | |
|  | Analysis of the full range of the p factor | | | | |
|  | Intraclass correlations (95% CI) | |  | Model-fitting estimates ^1^ (95% CI) | |
|  | MZ | DZ |  | Heritability (*h*^2^) | Nonshared environment |
| Observed p sum score | 0.45 (0.43, 0.47) | 0.14 (0.12, 0.16) |  | 0.41 (0.39, 0.43) | 0.59 (0.57, 0.61) |

Note. This sample contained 17,138 twin pairs, of which 5,129 monozygotic (MZ) and 12,009 dizygotic (DZ) twin pairs. MZ: monozygotic twins. DZ: dizygotic twins. ID: intellectual disability. Based on the number of standard deviations (= 0.60) beyond the mean value (= 0.19), observed p sum score = 2, ≥ 2, ≥ 3, and ≥ 4 correspond to mild ID (2-3.33 SD deviate from the mean), mild-profound ID (> 2 SD deviate from the mean), severe-profound ID (> 4.33 SD deviate from the mean), and profound ID (> 5.33 SD deviate from the mean), respectively. Observed p sum score was calculated as individuals’ total number of psychiatric diagnoses.

^1^Because the DZ correlation was less than half the MZ correlation, there was no indication of shared environmental effects (C) so we omitted it from the model.

**Supplementary Table 12.** Coefficients for familial coaggregation analysis regressing younger siblings’ standardized p factor score onto older siblings’ binned standardized p factor score (sensitivity analysis 4)

| Standardized p factor score | Beta (95% Confidence interval) |
| --- | --- |
| 0-1 SD | 0.04 (0.03, 0.06) |
| 1-2 SD | 0.11 (0.11, 0.12) |
| 2-3.33 SD | 0.22 (0.22, 0.23) |
| 3.33-4.33 SD | 0.33 (0.32, 0.34) |
| 4.33-5 SD | 0.36 (0.34, 0.38) |
| > 5 SD | 0.42 (0.38, 0.47) |

Note: Group without standardized p factor score less than the mean 0 was the reference group.

**Supplementary Figure 1.** Schematic of structural equation modeling diagram using latent p factor as outcome and p sum score as exposure

Bipolar disorder

Neuro

Psychiatric diagnosis

**Exposure:**

**Observed p sum score of *older siblings***

**Outcome:**

**Latent p score of *younger siblings***

Anxiety

Depression

Eating disorder

Autism

ADHD

Tics

Drug misuse

Alcohol abuse

Schizophrenia

Total number of the 10 psychiatric diagnoses of older siblings

Age of younger siblings

**Confounder**

Sub

Note. The arrow lines from latent factors to indicators with loadings ≤ 0.3 were omitted in the figure. Anxiety represents anxiety spectrum disorder including anxiety, obsessive-compulsive disorder, and post-traumatic stress disorder; ADHD is attention deficit hyperactivity disorder. Schizophrenia contains schizoaffective disorder; p factor represents the general psychopathology factor; Int represents specific internalizing factor; Sub represents specific substance misuse factor; Neuro represents specific neurodevelopmental factor.

**Supplementary Figure 2.** Distribution of the observed p sum score of older siblings

**
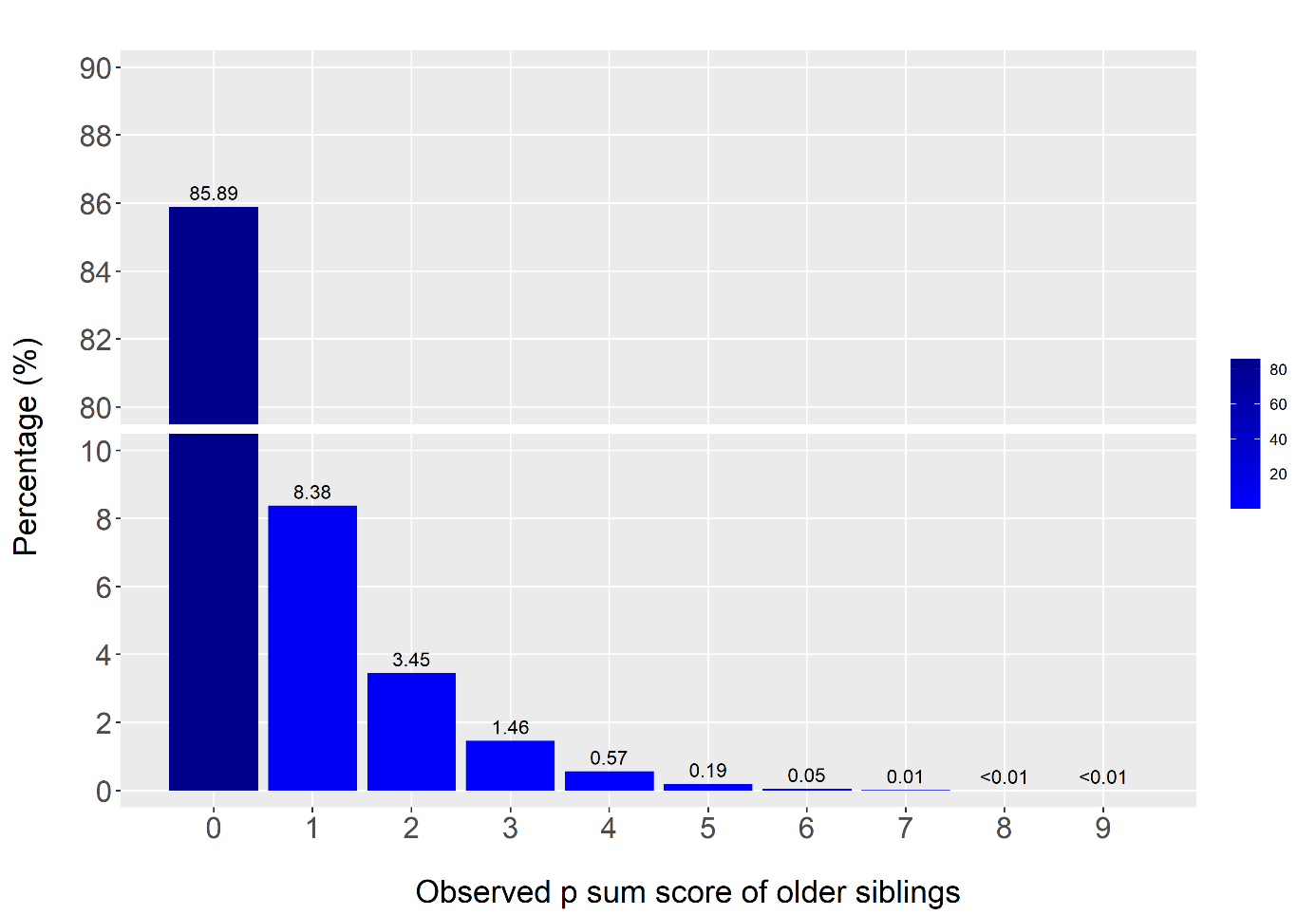
**

**Supplementary Figure 3.** Comparison of familial coaggregation results regressing younger siblings’ latent p factor onto older siblings’ binned observed p sum score (sensitivity analyses 1, 2, and 5) and intellectual disability (negative control analysis)


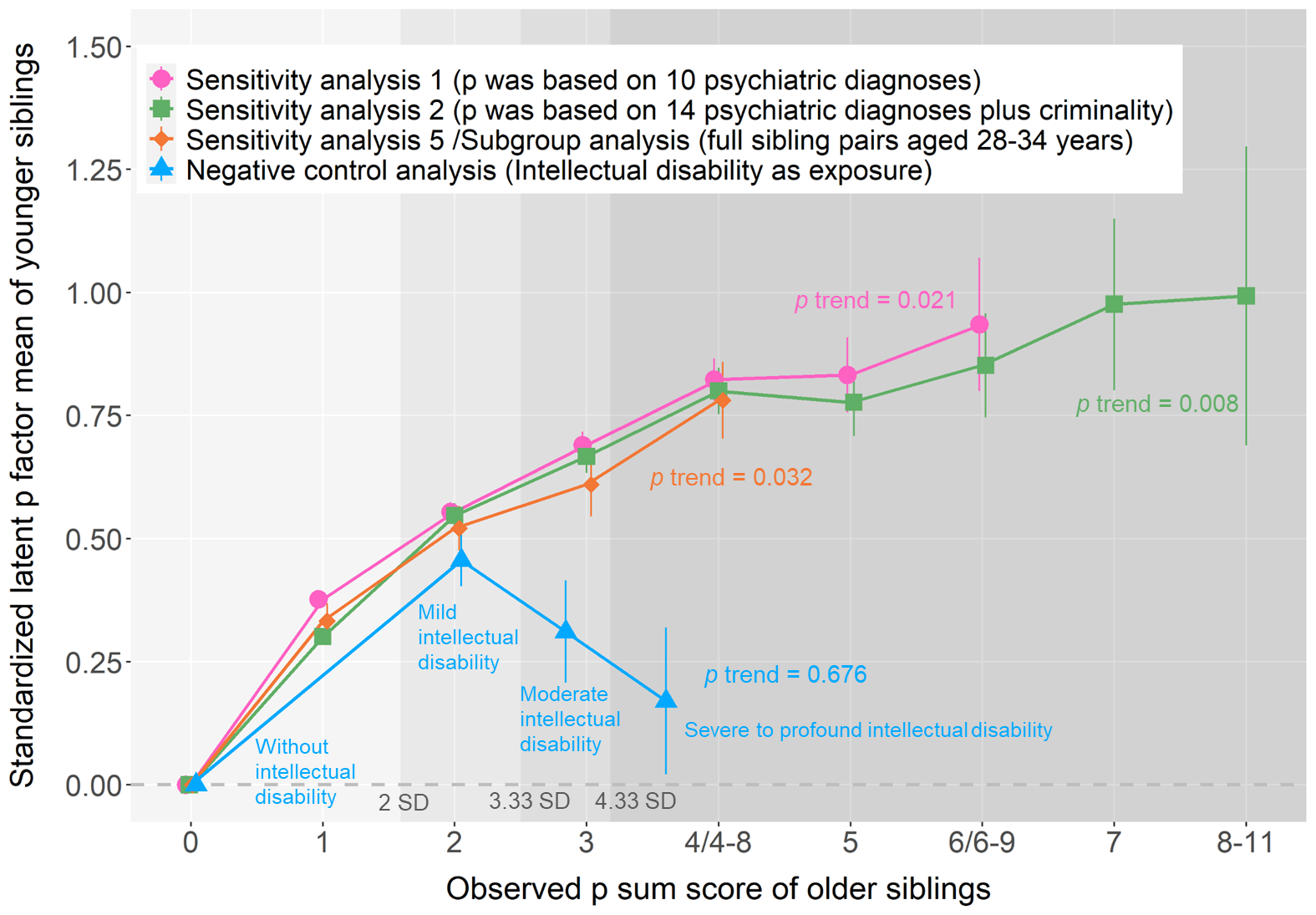


Note. The numbers (2 SD, 3.33 SD, and 4.33 SD) above the x axis represent number of standard deviation (SD) beyond the mean of the observed p sum score. Based on the deviations, the four regions with gradient shadings represent the severity of p factor in reference to the general population and correspond to “without”, “mild” (2-3.33 SD beyond the mean), “moderate” (3.33-4.33 SD beyond the mean), and “severe-profound” (> 4.33 SD beyond the mean) intellectual disabilities, respectively. The *p-*trend value was from linear-by-linear trend test (a significant *p*-trend value rejects the null hypothesis that the trend is non-linear). Bars represent 95% confidence intervals.

**Supplementary Figure 4.** Comparison of familial coaggregation results regressing younger siblings’ standardized p factor score onto older siblings’ standardized p factor score (sensitivity analyses 4) and intellectual disability (negative control analysis)


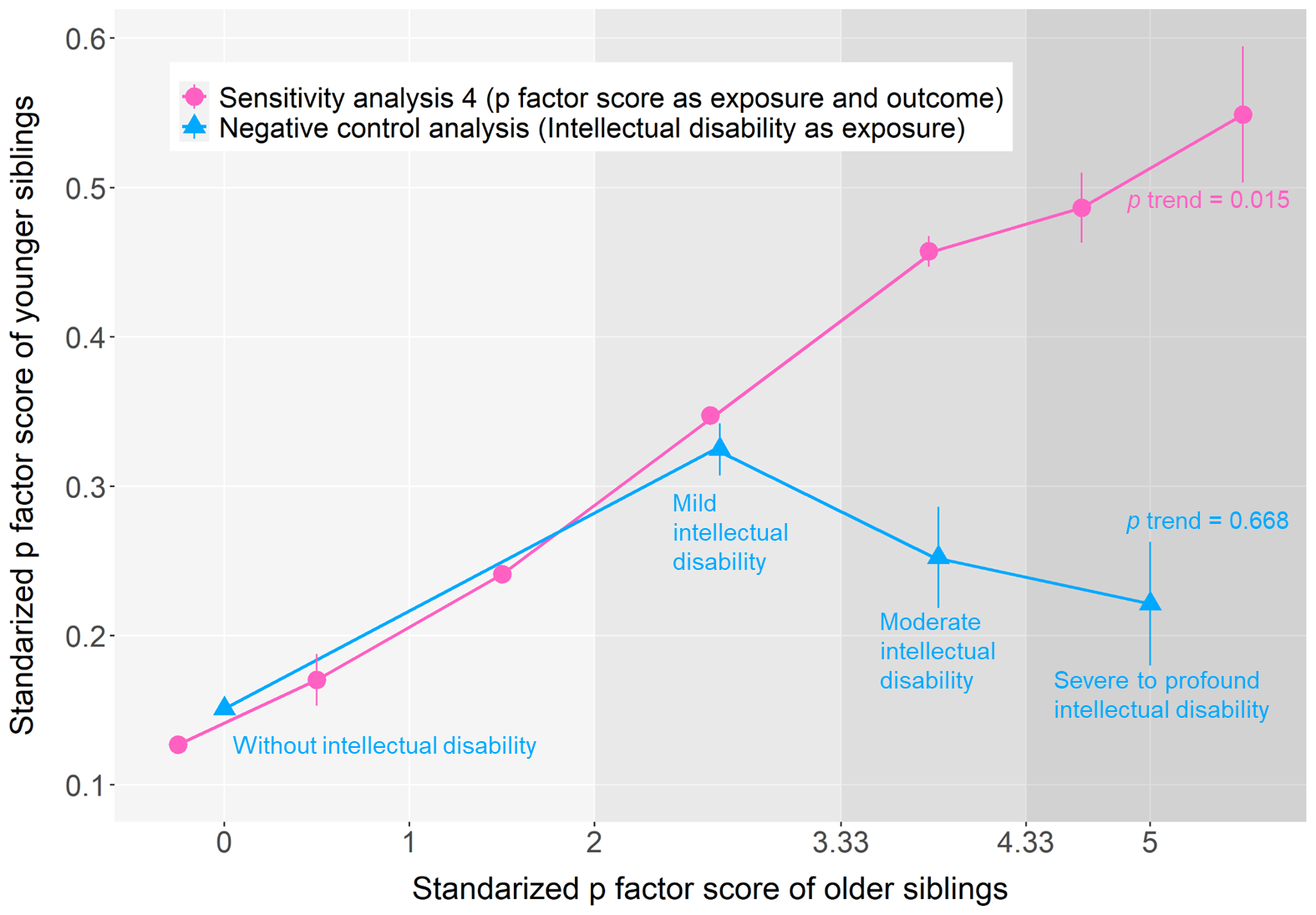


Note. Based on the deviations, the four regions with gradient shadings represent the severity of p factor score in reference to the general population and correspond to “without”, “mild” (2-3.33 SD beyond the mean), “moderate” (3.33-4.33 SD beyond the mean), and “severe-profound” (> 4.33 SD beyond the mean) intellectual disabilities, respectively. The *p-*trend value was from linear-by-linear trend test (a significant *p*-trend value rejects the null hypothesis that the trend is non-linear). Bars represent 95% confidence intervals.

**Supplementary References**

1. DeFries JC, Fulker DW. Multiple regression analysis of twin data. Behavior Genetics. 1985;15(5):467-473.

2. DeFries JC, Fulker DW. Multiple Regression Analysis of Twin Data: Etiology of Deviant Scores versus Individual Differences. Acta geneticae medicae et gemellologiae: twin research. 1988;37(3-4):205-216.

3. Plomin R, Kovas Y. Generalist Genes and Learning Disabilities. Psychological Bulletin. 2005;131(4):592-617.
